# Longitudinal Analysis of Substance Use as a Predictor PrEP Use and Adherence Among Young Transgender Women and Nonbinary Individuals

**DOI:** 10.1007/s10461-025-04986-3

**Published:** 2026-04-15

**Authors:** Alithia Zamantakis, Artur A. F. L. N. Queiroz

**Affiliations:** 1 Institute for Sexual and Gender Minority Health and Wellbeing, Northwestern University, Chicago, IL USA; 2 Psychiatry and Behavioral Sciences, Northwestern University, Chicago, IL USA

**Keywords:** Transgender persons, Substance use, Pre-exposure prophylaxis, Cohort studies, HIV

## Abstract

Previous research on the influence of substance use on PrEP uptake and adherence among transgender women has either relied on cross-sectional analyses or on combined samples of cisgender men who have sex with men and a much smaller subsample of transgender women. To the best of our knowledge, no longitudinal study has examined the relationship of substance use and PrEP use/adherence among young transgender women and nonbinary people assigned male at birth (TNBY). We sought to understand whether substance use (i.e., binge drinking, marijuana use, and illicit drug use) predicts PrEP uptake and adherence for TNBY over time. Using a sample of 242 young and racially/ethnically diverse TNBY from the RADAR cohort study, we performed bivariate and multivariate generalized estimating equations binary logistic regression to assess whether substance use predicted PrEP use and adherence and latent growth curve modeling to examine trends in self-reported PrEP use over time. There were no significant differences in PrEP use or adherence by race/ethnicity, gender or educational attainment. In multivariable models, binge drinking (OR 1.37, 95% CI 1.08–1.74), number of condomless sex partners (OR 1.08, 95% CI 1.04–1.13), and HIV/STI testing (OR 2.20, 95% CI 1.65–2.92) were all significantly positively associated with PrEP use in the past 6 months. Binge drinking (OR 1.27, 95% CI 0.97–1.67), number of condomless sex partners (OR 1.09, 95% CI 1.04–1.13), and HIV/STI testing (OR 1.85, 95% CI 1.35–2.53) were significantly positively associated with current PrEP use. Both age (OR 1.25, 95% CI 1.02–1.54) and binge drinking (OR 8.66, 95% CI 2.12–35.30) were significantly positively associated with PrEP adherence. Cannabis use was significantly negatively associated with PrEP adherence (OR 0.89, 95% CI 0.82–0.97). Latent growth curve modeling detailed that the proportion of participants using PrEP significantly increased over time across age, reducing initial differences in PrEP use by age. These findings provide valuable insights into the relationship between substance use and PrEP use and adherence, showing the different effects of binge drinking and problematic cannabis consumption on PrEP outcomes. These findings emphasize the importance of nuanced and intersectional approaches to HIV prevention, considering both demographic variability and behavioral health factors.

## Introduction

Pre-exposure prophylaxis (PrEP) is an evidence-based intervention found to reduce the likelihood of HIV acquisition by 99% when taken as directed [1, 2]. Originally designed as a daily oral pill in the forms of Truvada (emtricitabine/tenofovir disoproxil fumarate) and Descovy (emtricitabine/tenofovir alafenamide), PrEP also has evidence of efficacy when taken as a 2-1-1 regimen for cisgender men who have sex with men (i.e., 2 pills before sex followed by one pill every 24 h until 48 h after the last sex act [3]). PrEP is also available as a long-acting injectable (LAI) in the form of Apretude (cabrotegravir), as a bimonthly injection since 2021 [2]. Despite innovations to the type and dosing of PrEP, disparities in PrEP use remain among racially, ethnically, and gender minoritized populations [4–7].

Transgender women, in particular, have been found to be highly aware of PrEP, yet only 32% of transgender women surveyed in the National HIV Behavioral Surveillance System’s Transgender Cycle reported using PrEP [8]. Little research domestically has examined PrEP use among nonbinary individuals, or individuals whose gender identity is not encapsulated within the man/woman gender binary. Prior research in Canada has found nonbinary people assigned female at birth to be more aware of PrEP than transgender women [9]. The investigators also found that nonbinary individuals assigned male at birth reported the highest use of PrEP among transgender participants, while transgender women reported the lowest use.

Research indicates mixed findings regarding PrEP use among Black transgender women compared to their non-Black counterparts. Some studies suggest that Black transgender women are significantly less likely to report lifetime or current use of PrEP [10]. Conversely, other studies have shown that Black and/or Latina transgender women are more likely to report current PrEP use compared to white transgender women [8, 11]. Findings of higher reported PrEP use and initiation among Black and Latina transgender women are important as Black and Latina transgender women are more likely to be living with HIV than white transgender women [8, 12]. However, a high discontinuation rate across race/ethnicity highlights a need for further attentionto PrEP adherence [11].

Research on the relationship between substance use and PrEP use/adherence among transgender and nonbinary populations is limited and contrasting findings across studies have further limited our ability to understand the impact of substance use across the PrEP continuum for transgender and nonbinary people. For example, various studies have suggested that some forms of substance use (e.g., alcohol and stimulants) may increase PrEP use [10, 13, 14]. One study using structural equation modeling to examine the relationship between anti-transgender stigma, substance use, and HIV prevention found that anti-transgender stigma predicted increased substance use in the past twelve months [14]. Increased substance use was then positively associated with HIV prevention services, including PrEP use. However, a separate study that similarly found anti-transgenderstigma and minority stress to increase the likelihood of heavy substance use found heavy substance use to *decrease* intentions to use PrEP [15]. Thus, further research is needed to understand the impact of substance use across the PrEP continuum. [10, 13, 14, 16].

Thus far, a majority of the literature on PrEP and substance use has been focused on cisgender men who have sex with men (MSM) [17]. Of the research that does exist on substance use and PrEP among transgender women, most are limited to cross-sectional analyses, which may impact why findings have equivocated from study to study. Indeed, a recent review of transgender health research found that only 14% of studies published between 2020 and 2021 involved longitudinal analyses [18]. Longitudinal data and analyses are not only needed to identify and assess trends over time but to also assess causality [19]. As such, we sought to analyze the impact of substance use on PrEP use and adherence among young, transgender women and nonbinary people assigned male at birth (henceforth, TNBY) using data from the RADAR Cohort Study [20]. We hypothesized:

1. Consistent with prior literature, we hypothesized that rates of PrEP use and adherence would be greater among white TNBY in comparison with Black and Latine TNBY.
2. Alcohol, cannabis, and illicit drug use would be associated with increased odds of PrEP use over time.
3. Alcohol and illicit drug use would be associated with increased odds of PrEP adherence over time, while cannabis would be associated with decreased odds of PrEP adherence over time.

## Methods

### Participants

Data for these analyses were taken from RADAR, an ongoing longitudinal cohort study of young sexual and gender minorities assigned male at birth (AMAB) in Chicago who have sex with AMAB individuals, including young sexual minority men, transgender women, and nonbinary AMAB youth [20]. The primary objective of this cohort study is to apply a multilevel perspective to a syndemic of health issues associated with HIV and substance use. As described elsewhere, a subset of participants from two other cohorts of YMSM (i.e., Project Q2 and Crew 450) were recruited to the study in 2007 and 2010. A third and fourth cohort of participants were recruited and enrolled in the study beginning in 2015 and 2021, respectively [20, 21]. At the time of enrollment into their original respective cohorts, all participants were aged 16–20 years, assigned male at birth, spoke English, and had had a sexual encounter with a man in the previous year or identified as gay, bisexual, or transgender. Serious romantic partners of participants were also iteratively recruited into the cohort, so long as they were AMAB and under 29 years-of-age. Previously, participants were also able to refer up to 3 other AMAB individuals for enrollment between the ages of 16 and 29.

For the purposes of this study, we only included data from participants who ever endorsed a non-cisgender identity. We also excluded individuals who were younger than 18 prior to the FDA approving PrEP for individuals under 18 years old in 2018, resulting in a total sample size of n = 242. Study visits occurred every six months between 2015 and 2023 until a participant turned 30 years of age after which they switched to an annual visit schedule.

As gender identity may shift across the lifespan, particularly for youth and adolescents, we constructed a variable to capture consistent and fluid gender identification [22]. This included those who we termed “consistently non-cisgender.” These were individuals who, at baseline, identified as non-cisgender and who have continued to do so at all subsequent visits. Those who we termed “consistently fluid” reported non-cisgender and cisgender identities across multiple visits. Finally, two other groups included those who, at baseline, identified as non-cisgender but who have since identified as cisgender and those who, at baseline, identified as cisgender but who have since identified as non-cisgender.

### Procedures

Data were taken from both baseline and follow-up visits collected between February 2015 and October 2023. On average, participants contributed 8.8 visits of data to these analyses. Data were collected using computer assisted self-interview (CASI) software. All procedures were approved by the Northwestern University Institutional Review Board and participants were compensated for their involvement.

### Measures

#### Demographics

At baseline, participants reported demographic information, including age, sexual identity, educational attainment, housing status, race, ethnicity, and gender. We constructed an age-time interaction variable, as well, to investigate whether there was an interaction between age and time. Participants were asked their gender again at following visits. We constructed an additional variable, “gender category” to track changes in cisgender/transgender identity across time. This included those who were consistently non-cisgender (i.e., they endorsed a gender other than the sex they were assigned at birth at each visit), change cisgender to non-cisgender (i.e., at earlier visits, they identified as cisgender, but that changed in later visits), change non-cisgender to cisgender (i.e., at earlier visits, they identified as a gender other than the sex they were assigned at birth, but that changed in later visits), and fluid (i.e., participants consistently alternated between a cisgender identity and non-cisgender identity across multiple visits).

#### PrEP Use

PrEP use was measured in two ways. Participants were asked to report whether they had taken any form of PrEP (at time of survey, this only included oral PrEP) in the past six months to reduce their risk of HIV transmission. Participants could endorse “yes” or “no.” Participants were also asked if they are currently taking any form of PrEP to reduce their risk of HIV transmission. Again, participants could endorse “yes” or “no.”

#### PrEP Adherence

Participants were asked whether they had missed any doses of oral PrEP in the past seven days. If yes, they were asked whether they missed a dose for each of the 7 days prior to survey administration. Those who reported PrEP use for four or more of the last 7 days were coded as PrEP adherent. Those who reported missing more than 3 doses of PrEP in the last 7 days were coded as non-adherent.

#### Alcohol Use

The Alcohol Use Disorders Identification Test (AUDIT), a 10-item problematic drinking screener [23], was used to measure alcohol use. Example items included, “How many drinks containing alcohol do you have on a typical day when drinking?” “How often do you have six or more drinks on one occasion?” “Has a relative, friend, doctor or other health care worker been concerned about your drinking or suggested you cut down?” Participants were asked to answer based on the past six months. AUDIT scores range from 0 to 40, with 0–7 classifying as low risk and 20–40 as high risk or almost certainly dependent on alcohol. Binge drinking was measured based on one of the items from AUDIT: “How often do you have six or more drinks on one occasion in the past six months?” Participants could select from never, less than monthly, monthly, weekly, and daily or almost daily. If a participant endorsed anything other than ‘never,’ they were coded as engaging in binge drinking in the past six months. To account for the combined effect of alcohol use frequency and quantity, we also included a quantity*frequency interaction. This interaction was created by multiplying two AUDIT items. The first asked, “How often do you have a drink containing alcohol?” Responses included never (0), monthly or less (1), 2–4 times per month (2), 2–3 times per week (3), and 4 or more times per week (4). The second asked, “How many drinks containing alcohol do you have on a typical day when you are drinking?” Responses included 0 (0), 1 or 2 (1), 3 or 4 (2), 5 or 6 (3), 7 to 9 (4), and 10 or more (5). Interaction scores ranged from 0 to 20, with 20 signifying high frequency drinking of a high quantity of alcohol.

#### Marijuana Use

Marijuana use was also measured in two ways. Participants were asked whether they had used marijuana in the past six months at each visit. The Cannabis Use Disorder Identification Test (CUDIT-R), an 8-item screening instrument used to identify problematic marijuana use [24], was administered to participants who endorsed that they had used marijuana in the past six months. CUDIT-R is an adaptation from the 10-item CUDIT, which was based on AUDIT [23, 25]. Example items included, “How often do you use marijuana?” “How many hours were you ‘stoned’ on a typical day when you had been using marijuana?” “How often during the past 6 months did you fail to do what was normally expected from you because of using marijuana?” CUDIT-R scores range from 0 to 32, with scores of 12 or more indicating a possible cannabis use disorder.

#### Illicit Drug Use

Illicit drug use included participant endorsement of using cocaine, heroin, methamphetamine, GHB (gamma-hydroxybutyrate), ketamine, LSD (lysergic acid diethylamide), or MDMA (3,4-Methylenedioxy methamphetamine) in the past six months at each study visit. Participants who endorsed using one or more of the above drugs were coded as engaging in illicit drug use.

Sexual risk behavior. The number of condomless anal or vaginal sex partners a participant had in the prior six months was derived from the HIV Risk Assessment of Sexual Partnerships (H-RASP) [26]. Data was winsorized at three standard deviations from the mean to reduce the impact of extreme values.

HIV/STI testing. To assess HIV and STI testing practices, participants were asked “In the past 6 months, how many times have you been tested for HIV?” and “In the past 6 months, have you been tested for any STI?”. A binary variable was derived based on whether participants had tested for either HIV or STIs (1) or neither (0) in the past 6 months.

### Statistical Analyses

First, descriptive statistics were used to describe the demographic characteristics of participants (n = 242) at their baseline and most recent visit as well as to describe HIV prevention and substance use behaviors across all study visits (n = 2044). Using generalized estimating equations (GEE) with logit link for binary outcomes and an exchangeable working correlation structure, we examined whether individual demographic characteristics, d substance use behaviors, sexual risk behavior, and recent HIV/STI testing predicted past six-month PrEP use, current PrEP use, and PrEP adherence at the subsequent six-month follow-up visit. Variables that were found to be statistically significant predictors (*p* < 0.10) in bivariable GEE analyses were included in multivariable GEE analyses controlling for demographics characteristics.

Finally, latent growth curve modeling was conducted using Mplus to examine trends in self-reported PrEP use between 2015 and 2023. To account for differing participant assessment schedules, individually varying time scores were used to model trends based on time since January 1st, 2015. Maximum likelihood estimation was used to address missing data. Model fit was assessed using Akaike Information Criterion (AIC), Bayesian Information Criterion (BIC), and adjusted BIC. Latent growth curves were first analyzed without covariates to identify the best fitting model. An intercept only, intercept plus linear slope, and intercept plus linear slope and quadratic effect models were compared. After establishing the best-fitting model, age and racial/ethnic identity were added to the model as covariates to examine their effect on the growth terms (intercept, slope, and quadratic).

## Results

### Descriptive Data on Analytic Sample

Participants contributed 2044 unique visits of data to this study, an average of 8.8 visits per participant (IQR: 9.0). On average, participants missed 3.7 visits across eight years (2015–2023; SD = 3.6), for an average of 26.7% of visits missed (SD = 24.9%). 16.5% of participants were lost to follow-up after having not completed a visit after 3 years.

See Table 1 for full demographics of the analytic sample. Approximately 41% (n = 99) of participants consistently identified as non-cisgender, while the remaining 59% previously endorsed a cisgender identity but most recently endorsed a non-cisgender identity (n = 80), previously endorsed a non-cisgender identity and most recently endorsed a cisgender identity (n = 7), or were consistently fluid in endorsing a cisgender/non-cisgender identity (n = 56). Participants endorsed a range of gender identities, including man (n = 44), woman (n = 48), transgender (n = 18), gender nonconforming (GNC; n = 16), genderqueer (n = 22), and nonbinary (n = 71) at the most recent study visit. The study population encompassed a diverse range of racial and ethnic backgrounds. The largest racial/ethnic group was non-Hispanic White (34.3%), followed by Hispanic/Latine (29.8%) and non-Hispanic Black (24.4%). Non-Hispanic Other accounted for 11.6% of participants.

**Table 1:** Participant characteristics (n = 242)

	First visit		Most recent visit	
	n	%	n	%
*Gender category*				
Consistently non-cisgender	99	40.9		
Change cisgender to non-cisgender	80	33.1		
Change non-cisgender to cisgender	7	2.9		
Fluid	56	23.1		
*Gender*				
Man	128	52.9	44	18.2
Woman	15	6.2	48	19.8
Transgender	40	16.5	18	7.4
Gender nonconforming	7	2.9	16	6.6
Genderqueer	10	4.1	22	9.1
Nonbinary	21	8.7	71	29.3
Other	21	8.7	23	9.6
*Race/ethnicity*				
Non-Hispanic Black	59	24.4		
Hispanic/Latinx	72	29.8		
Non-Hispanic white	83	34.3		
Non-Hispanic other	28	11.6		
*Education*				
<High school graduate	36	14.9	18	7.4
High school graduate or GED	74	30.6	48	19.8
Some college or trade school	115	47.5	90	37.2
College graduate	17	7.0	86	35.5
*Unstable housing*				
No	224	92.6	231	95.5
Yes	18	7.4	11	4.5
*Sexual identity*				
Gay	106	43.8	55	22.7
Bisexual	38	15.7	38	15.7
Straight	14	5.8	26	10.7
Queer	42	17.4	72	29.8
Other	42	17.4	51	21.1

	M	SD	M	SD
Age (in years)	20.9	2.6	25.3	3.9

Gender identity shifted between the first visit and most recent visit. At first visit, nearly 53% of the sample (n = 128) identified as a man compared to just over 18% (n = 44) at the most recent visit. At first visit, nearly 9% (n = 21) identified as nonbinary compared to over 29% at the most recent visit (n = 71). Sexual orientation also shifted between the two visits. At first visit, nearly 44% (n = 106) identified as gay compared to just under 23% at the most recent visit (n = 55). The number of participants identifying as bisexual remained stable across time (n = 38 at both time points). Only a minority reported experiencing unstable housing (7.4% at first visit, 4.5% at most recent visit). See Table 2 for descriptive statistics of study variables. Averaging across all time points, participants reported using PrEP in the past six months at just over 25% of all visits (n = 517). However, participants reported they were currently using PrEP at just over 19% of all visits (n = 393), indicating some discontinuation within each wave. Most participants reported taking PrEP as an oral pill across study visits (n = 384; 98.0%). Participants reported HIV/STI testing at 1566 visits (76.6% of all visits). The median number of partners with which participants had condomless anal/vaginal sex in the past six months was 1 (IQR: 2).

**Table 2:** Descriptive statistics of HIV prevention and substance use variables by study visit (n = 2093)

	n	%
PrEP Use in past 6 months	517	25.3
Current PrEP user	393	19.2
Adherent to PrEP^1^	339	96.0
PrEP Mode^2^		
Pill	384	98.0
Injection	8	2.0
Binge drink in past 6 months	1127	55.1
Marijuana use in past 6 months	1482	72.5
Illicit drug use in past 6 months^3^	590	28.9
HIV/STI testing in past 6 months	1566	76.6

	Median	IQR
AUDIT sum score (range 0–40)	4	6
CUDIT sum score (range 0–32)	5	11
Alcohol quantity × Frequency (range 0–20)	2	3
Condomless anal/vaginal sex partners in past 6 months	1	2

^1^Percentages are calculated with the number of current PrEP users as the denominator. There is missingness due to adherence questions not being administered at all visits

^2^Percentages are calculated with the number of current PrEP users as the denominator

^3^Includes cocaine, heroin, methamphetamine, GHB, ketamine, LSD, and MDMA

Averaging across all time points, participants reported binge drinking in the past six months at 55.1% of all study visits (n = 1127), marijuana use in the past six months at 72.5% of all visits (1482), and illicit drug use in the past six months at 28.9% of all visits (n = 590). The median AUDIT sum score across all time points was 4 (IQR: 6), signifying low risk of alcohol dependence. The median CUDIT sum score across all time points was 5 (IQR: 11), signifying low risk of a cannabis use disorder. Finally, the median alcohol quantity-frequency interaction score across all time points was a 2 (IQR: 3), signifying lower frequency of a lower quantity of alcohol.

### Bivariate Results

Time, age, cisgender identity, binge drinking in the past 6 months, marijuana use in the past 6 months, number of condomless anal or vaginal sex partners in the past 6 months, and HIV/STI testing in the past 6 months were all found to have significant associations with current PrEP use, as well. Current PrEP use increased over time, and respondents currently endorsing a cisgender identity were significantly less likely to report current PrEP use than those currently endorsing a non-cisgender identity.

Binge drinking was significantly associated with increased odds of being adherent to PrEP while CUDIT-R score was significantly associated with decreased odds of being adherent to PrEP.

A summary of bivariate GEE binary logistic regression models can be found in Table 3. Analyses found a significant positive relationship between PrEP use in the past 6 months and time, age, current cisgender identity, race/ethnicity, binge drinking in the past 6 months, marijuana use in the past 6 months, illicit drug use in the past 6 months, number of condomless anal or vaginal sex partners in the past 6 months, and HIV/STI testing in the past 6 months. Non-Hispanic Black participants were significantly more likely to endorse PrEP use in the past 6 months than non-Hispanic white participants. Participants currently endorsing a cisgender identity were significantly less likely to report PrEP use in the past 6 months than those currently endorsing a non-cisgender identity.

**Table 3:** Summary of bivariate GEE binary logistic regression models

	PrEP use past 6 months				Current PrEP use				PrEP adherence			
	OR	95% OR		*p*-value	OR	95% OR		*p*-value	OR	95% OR		*p*-value
Time	1.09	1.02	1.17	**0.007**	1.09	1.02	1.17	**0.012**	0.97	0.76	1.24	0.798
Age	1.09	1.03	1.16	**0.002**	1.10	1.04	1.16	**0.002**	1.11	0.92	1.35	0.266
Cisgender^1^	0.72	0.53	0.97	**0.030**	0.75	0.54	1.02	**0.071**	0.91	0.34	2.45	0.854
Consistently not cisgender^2^	0.94	0.59	1.50	0.798	0.86	0.52	1.43	0.563	1.30	0.34	5.01	0.702
Greater than HS grad^3^	1.28	0.91	1.81	0.158	1.39	0.92	2.09	0.115	0.80	0.23	2.86	0.735
Race/Ethnicity^4^												
Non-Hisp. Black	1.82	1.02	3.25	**0.044**	1.61	0.85	3.06	0.145	1.63	0.29	9.25	0.582
Hisp./Latinx	1.14	0.65	1.99	0.648	1.13	0.60	2.11	0.711	1.34	0.33	5.48	0.684
Non-Hisp. Other	1.45	0.69	3.06	0.327	1.35	0.58	3.12	0.487	0.42	0.10	1.85	0.250
Binge drink in past 6 months^2^	1.35	1.07	1.71	**0.013**	1.26	0.96	1.67	**0.098**	2.98	0.98	9.06	**0.055**
Marijuana use in past 6 months^2^	1.34	0.95	1.88	**0.092**	1.37	0.96	1.96	**0.081**	0.56	0.13	2.33	0.423
Illicit drugs use in past 6 months^2^	1.39	1.08	1.79	**0.010**	1.24	0.92	1.67	0.150	1.38	0.43	4.50	0.589
Alcohol quantity × frequency	1.02	0.97	1.06	0.512	1.03	0.98	1.08	0.194	1.09	0.84	1.41	0.528
AUDIT sum score	1.01	0.99	1.04	0.308	1.02	0.99	1.04	0.245	1.13	0.94	1.37	0.200
CUDIT sum score	1.01	0.99	1.04	0.260	1.01	0.98	1.03	0.647	0.94	0.89	1.01	**0.079**
Number of condomless anal/vaginal sex partners	1.13	1.07	1.19	**<.001**	1.11	1.06	1.17	**<.0001**	0.98	0.88	1.09	0.713
Tested for HIV/STIs in past 6 months 5^5^	2.30	1.76	3.00	**<.0001**	1.96	1.45	2.64	**<.0001**	–	–	–	–

Bolded *p*-value = significant at ≤ 0.05, ≤ 0.01, ≤ 0.001

^1^Reference is non-cisgender

^2^Reference is no

^3^Reference is high school graduate or less

^4^Reference is non-Hispanic white

^5^PrEP adherence model failed to converge due to sparse or missing outcome-predictor combinations, leading to unstable parameter estimation

### Multivariate Results

**Fig. 1:**
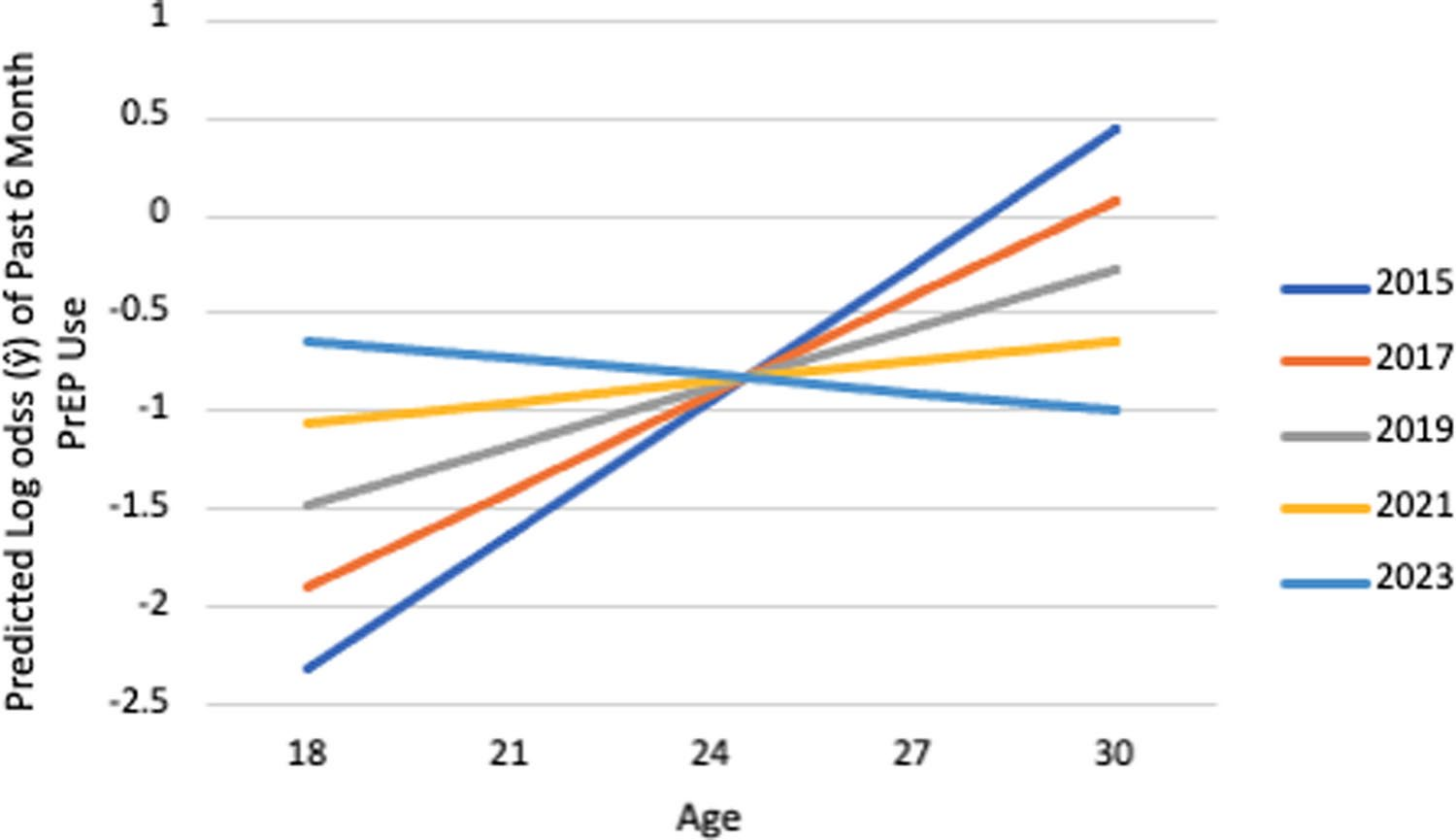
Predicted log odds of PrEP use in the past 6 months by age and year

**Fig. 2:**
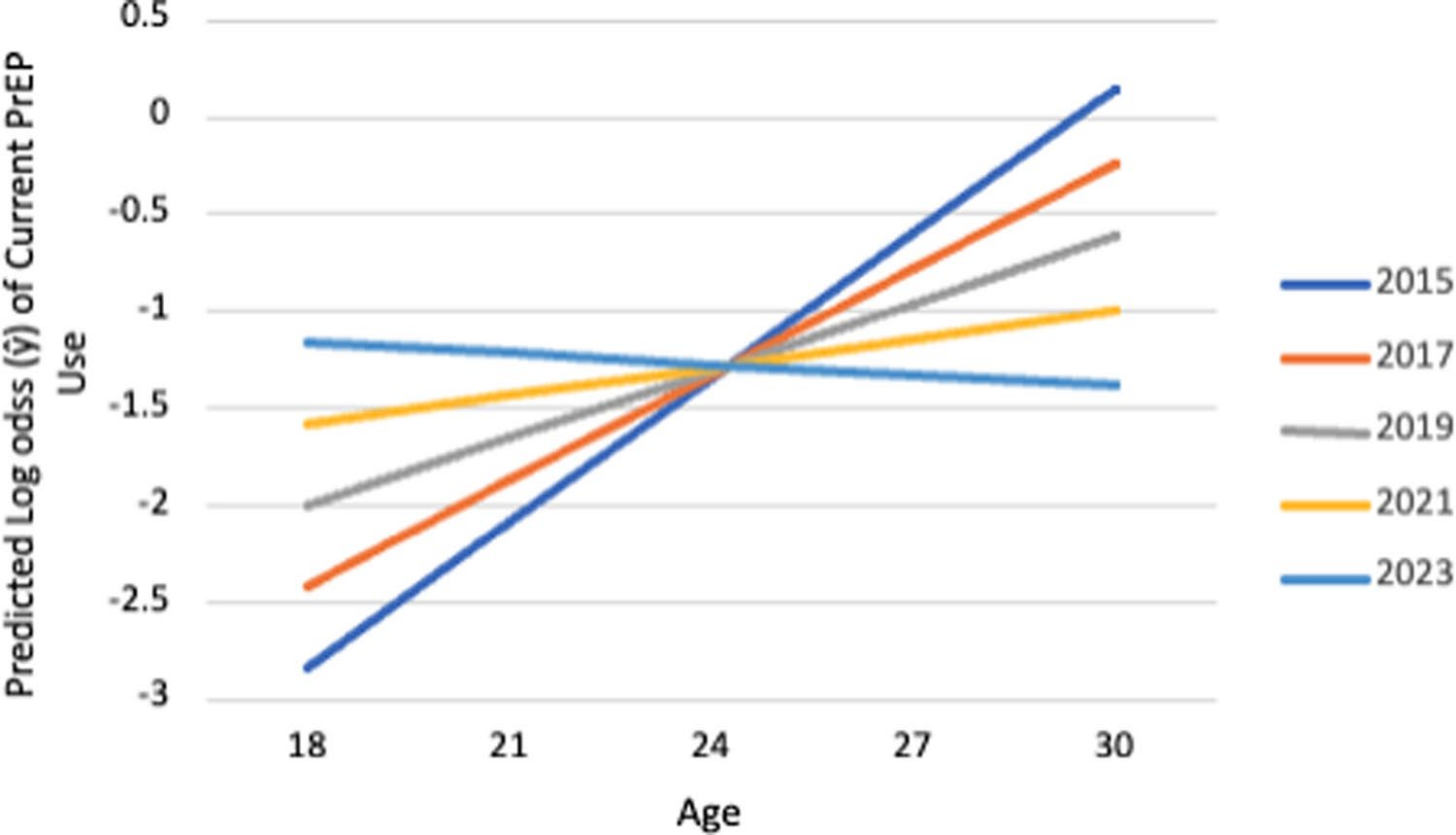
Predicted log odds of current PrEP use by age and year

Analyses identified significant positive associations between PrEP use in the past 6 months, binge drinking in the past 6 months, number of condomless anal or vaginal sex partners in the past 6 months, and HIV/STI testing in the past 6 months. Number of condomless anal or vaginal sex partners in the past 6 months and HIV/STI testing in the past 6 months were also both significantly associated with increased odds of being a current PrEP user. The age-time interaction was significantly inversely associated with current PrEP use and PrEP use in the past 6 months. The interaction shows a greater age discrepancy in current PrEP use and PrEP use in the past 6 months, with older participants more likely than younger participants to be using PrEP, at the start of RADAR. However, the age discrepancy diminishes over time (see Figs. 1, 2). Respondents who reported binge drinking in the past 6 months were significantly more likely to adhere to PrEP than those who did not report binge drinking. Additionally, a significant inverse association was observed between CUDIT-R sum scores and PrEP adherence, indicating that higher cannabis use was linked to lower adherence. Detailed results from the multivariate generalized estimating equations (GEE) binary logistic regression models are presented in Table 4**.**

**Table 4:** Summary of multivariable GEE binary logistic regression models

	PrEP use past 6 months				Current PrEP use				PrEP adherence			
	OR	95% OR		*p*-value	OR	95% OR		*p*-value	OR	95% OR		*p*-value
Time	1.05	0.97	1.14	0.264	1.03	0.95	1.13	0.452				
Age	1.05	0.98	1.12	0.182	1.07	1.00	1.14	0.055	1.25	1.02	1.54	**0.032**
Age*Time	0.97	0.95	0.99	**0.007**	0.97	0.95	0.99	**0.014**				
Cisgender^1^	0.91	0.66	1.26	0.574	0.96	0.68	1.35	0.814	0.75	0.26	2.19	0.601
Greater than HS grad^2^	1.03	0.68	1.54	0.902	1.12	0.73	1.73	0.602	0.32	0.08	1.26	0.103
Race/Ethnicity^3^												
Non-Hisp. Black	1.72	0.92	3.22	0.090	1.49	0.75	2.95	0.254	1.79	0.23	14.25	0.580
Hisp./Latinx	1.09	0.63	1.91	0.751	1.10	0.59	2.05	0.753	2.01	0.48	8.45	0.340
Non-Hisp. other	1.09	0.46	2.60	0.848	0.83	0.28	2.50	0.744	0.35	0.07	1.64	0.182
Binge drink in past 6 months^2^	1.37	1.08	1.74	**0.010**	1.27	0.97	1.67	0.085	8.66	2.12	35.30	**0.003**
Marijuana use in the past 6 months^2,5^	1.25	0.88	1.78	0.213	1.32	0.92	1.90	0.133				
Illicit drugs use in past 6 months^2,4^	1.09	0.84	1.40	0.526								
CUDIT sum score^5^									0.89	0.82	0.97	**0.007**
Number of condomless anal/vaginal sex partners^6^	1.08	1.04	1.13	**0.001**	1.09	1.04	1.13	**<.001**				
Tested for HIV/STIs in past 6 months^6^	2.20	1.65	2.92	**<.0001**	1.85	1.35	2.53	**<.001**				

Bolded *p*-value = significant at ≤ 0.05, ≤ 0.01, ≤ 0.001

^1^Reference is non-cisgender

^2^Reference equals no

^3^Reference is non-Hispanic white

^4^Not included for current PrEP use and PrEP adherence because it was non-significant in bivariate analyses

^5^Not included for PrEP use in past 6 months and current PrEP use because it was non-significant in bivariate analyses

^6^Not included for PrEP adherence because it was non-significant in bivariate analyses

### Latent Growth Curve Modeling

Latent growth curve modeling was conducted to further examine trends in self-reported PrEP use between 2015 and 2023. Model fit statistics for the three-step latent growth modeling approach are presented in Table 5. The best fitting model included the intercept, linear slope, and quadratic effect as it produced the lowest AIC, BIC, and adjusted BIC statistics. Linear effect of time on PrEP use was statistically significant (β = 0.99, *p* = 0.015) indicating the proportion of participants using PrEP increased with time. Additionally, the quadratic effect of time on PrEP use was statistically significant (β = −0.08, *p* = 0.037) indicating the magnitude of the increase in PrEP use diminished over time. There were no statistically significant (*p* > 0.05) differences in intercept, linear slope or quadratic effect based on participants’ age or race/ethnicity.

**Table 5:** Model fit statistics for PrEP use latent growth analyses in a sample of gender minorities (n = 242)

	AIC	BIC	Adjusted BIC
Intercept	1546.431	1553.409	1547.069
Intercept + linear slope	1477.820	1495.265	1479.416
Intercept + linear Slope + quadratic	1442.556	1473.960	1445.431

## Discussion

This manuscript is among the first to longitudinally analyze the relationship of substance use with PrEP use and adherence over time among TNBY. Among a racially, sexually, and gender diverse cohort of TNBY, we found high rates of reported binge drinking, marijuana use, and illicit drug use in the past six months. Twenty-eight percent of our sample reported illicit drug use (i.e., cocaine, heroin, methamphetamine, GHB, ketamine, LSD, and MDMA). Cocaine, heroin, methamphetamine, and hallucinogen use are much lower among young adults in the general population (3.1%, 0.1%, 0.3%, and 6.7%, respectively). This highlights a need for greater attention to interventions targeting illicit drug use among TNBY [27]. For example, interventions like Keep It Up!, a digital health intervention for young men who have sex with men (YMSM) that addresses PrEP use and substance use, can be adapted for TNBY populations [28].

Binge drinking in the past six months, number of condomless anal or vaginal sex partners in the past 6 months and HIV/STI testing in the past 6 months emerged as significant predictors of PrEP use, with the latter two also emerging as significant predictors of current PrEP use. Consistent with prior literature across various populations, our study found that current PrEP use andPrEP use in the past six months increased with age [29–31]. However, the differences in age for PrEP use decreased over time, as indicated by the negative age-time interaction. Our analysis also corroborated previous findings that binge drinking and illicit drug use significantly predicted PrEP use [13, 32, 33]. Notably, binge drinking was associated with much higher PrEP adherence, with PrEP adherence having greater odds of being reported by those who reported binge drinking. It is possible that this association between binge drinking and PrEP use/adherence is due to risk-compensation, with individuals who binge drink knowing their greater vulnerability to the acquisition of HIV and thus ensuring their use of and adherence to biomedical HIV prevention [34, 35]. This is complicated, though, by AUDIT sum score not significantly predicting PrEP use or adherence and illicit drug use not predicting PrEP adherence in contrast to prior findings [13, 14]. Importantly, though, this study bridges existing scientific evidence by demonstrating that these predictors of PrEP use and adherence, previously established in other populations, also apply to non-cisgender individuals. This innovative finding highlights the broader applicability of these predictors and underscores the need for inclusive public health strategies that address the unique needs of diverse populations.

Also of note, current PrEP use and PrEP use in the past 6 months were both significantly associated with number of condomless sex partners and HIV/STI testing in the past 6 months. This suggests, similarly to binge drinking, that TNBY in Chicago may be aware of their increased vulnerability to HIV acquisition, resulting in their increased use of PrEP.

While increases in age predicted much greater current PrEP use in the initial years of RADAR (2015–2019), this began to shift in 2021. Over time, the differences in age decreased such that those younger age predicted higher current PrEP use among those enrolling in RADAR in 2023. This highlights the likely impact of increased public health campaigns and interventions targeting young adults and teens for PrEP use.

Of note, there were no significant differences by race/ethnicity for current PrEP use, PrEP use in the past six months, or PrEP adherence. We originally thought this may be due to the work of public health researchers and practitioners, as well as campaigns targeting Black sexual and gender minorities in Chicago that may have had an impact in reducing disparities in PrEP care. PrEP4Love, for example, was a public health campaign targeting Black, gay, bisexual, and other men who have sex with men, transgender women of color, and Black, cisgender, heterosexual women [36]. However, another analysis of PrEP use over time among TNBY and young, cisgender men who have sex with men (YMSM) in the RADAR cohort study found PrEP use to be increasing at a greater rate among non-Hispanic white and Hispanic/Latine participants than among Black participants [37]. While some prior research documents lower PrEP use among non-Hispanic Black TNBY in comparison to non-Hispanic white TNBY [8, 10, 38, 39], other research has found the opposite [8, 11]. In contrast to this prior research, which relies on cross-sectional data, our manuscript is a longitudinal analysis of a cohort of TNBY. Further research is needed to understand the lack of significant differences in PrEP use or adherence by race/ethnicity over time in Chicago for TNBY. The findings in this manuscript are particularly important considering disparities in substance use among transgender and nonbinary youth and young adults. Previous research has documented higher odds of problematic alcohol use, heavy episodic drinking, cigarette smoking, problematic cannabis use, stimulant and illicit drug use, vaping, binge drinking, and polysubstance use among transgender and nonbinary individuals, including youth, in comparison to cisgender heterosexual individuals and cisgender sexual minorities [40–44]. Further, disparities within transgender populations have documented greater odds of reported alcohol use, cigarette smoking, and stimulant use among transgender and nonbinary individuals assigned male at birth in comparison to those assigned female at birth [40], as well as more frequent binge drinking among racially minoritized transgender and nonbinary young adults than among those who are white [44]. This research has also documented the association between increased substance use and denial of health care, invasive questions from health care providers, and verbal harassment from health care providers, as well as the role of transgender victimization in mediating the relationship between gender identity and increased substance use [41, 43].

Researchers have found, though, that access to gender affirming care may mitigate the impacts of minority stress and discrimination on transgender and nonbinary individuals’ substance use [15]. As gender affirming care is also associated with PrEP use and adherence [45], this highlights the increasing need to integrate gender affirming care, HIV prevention, and substance use treatment/prevention. Unfortunately, increasing political attacks have resulted in diminished access to gender affirming care for young adults and teens [46], which will have far ranging impacts on transgender youth and young adults’ mental health [47, 48], and based on this body of research, their use of substances [15], and HIV prevention interventions [45].

These findings should be considered in light of the following limitations. First, PrEP adherence was generally high across time, with two exceptions: a significant drop in adherence among non-Hispanic other race/ethnicity TNBY in 2020 during the pandemic and a more recent significant drop in adherence among non-Hispanic white TNBY in 2023. Regardless of fluctuations, most recently reported adherence was 96% across the cohort. Further research may be warranted among a cohort with greater variance in PrEP adherence. Second, all measures were self-report, which may be subject to bias due to social desirability, recall error, or misunderstanding of items. The RADAR cohort study, though, does include both self-report and point-of-care testing for PrEP use and adherence. Prior research has found more accurate self-reporting in the presence of such testing [49]. Finally, while we have included number of condomless sex partners in the past 6 months as a measure within these analyses, it is not possible to know from this whether the condomless sex partners were monogamous, long-term partners with whom it would be “safer” to not use a condom. Similarly, while we have included HIV/STI testing in the past 6 months and found a high frequency of testing (i.e., testing was reported at greater than 76% of all visits), this could also be a result of participants engaging in testing as part of the RADAR cohort study.

## Conclusion

Our study highlights several significant trends in PrEP use and adherence across a diverse cohort, with key insights into how demographic factors and substance use behaviors impact HIV prevention efforts. Notably, shifts in gender identity and sexual orientation over time point to the fluidity of identity among participants, which may influence their healthcare needs and behaviors. The positive associations between binge drinking, condomless sex, and HIV/STI testing and PrEP use suggest that tailored prevention strategies are essential for specific populations. Furthermore, the inverse relationship between cannabis use and PrEP adherence underscores the need for targeted interventions to support adherence in individuals with higher substance use. These findings emphasize the importance of nuanced and intersectional approaches to HIV prevention, considering both demographic variability and behavioral health factors.

## Data Availability

Data from this analysis, as well as the first four waves of data collection from the RADAR Cohort Study, are available for public use through the Inter-University Consortium for Political and Social Research (ICPSR) website. This includes both public-use files, which are available to anyone for download and use, and restricted-use files to which access is only granted following an application process and agreement by researchers to adhere to strict requirements for maintaining data confidentiality.
